# Ethnic and Age Disparities in Patients Taking Long-acting Injectable Atypical Antipsychotics

**DOI:** 10.7759/cureus.1772

**Published:** 2017-10-12

**Authors:** Mateen Soleman, Nikki Lam, Benjamin K Woo

**Affiliations:** 1 Western, Western University; 2 College of Medicine, Northeast Ohio Medical University (NEOMED); 3 UCLA

**Keywords:** long-acting injections, antipsychotics, racial disparities, duration of untreated psychosis, asian americans, schizophrenia, inpatient psychiatry, psychopharmacology

## Abstract

Introduction

This study will determine whether different ethnicities and different age groups receive equal amounts of long-acting atypical antipsychotics in comparison to their oral equivalents.

Methods

Secondary analyses of data from the Los Angeles County Department of Health Services Electronic Health Record (total N=63,134 inpatient visits) were performed. Chi-squared statistics were used to compare ethnicity and age with the use of either risperidone injectable or paliperidone palmitate (r-LAIs) versus risperidone oral.

Results

Among the 63,134 total inpatient visits, there were 3,011 patient visits that included the use of an atypical antipsychotic. Of these 3,011 visits, 452 (15.0%) were on r-LAIs and 2,559 (85.0%) were on risperidone oral. No statistically significant disparities were identified with the use of r-LAIs as compared to oral risperidone amongst ethnic groups (chi-square = 0.88, df = 3, p = 0.831). However, there was a statistically significant difference with the use of r-LAIs as compared to oral Risperidone amongst age groups, favoring younger patients (chi-square = 13.46, df = 3, p < 0.004).

Conclusion

Our data indicate a lack of ethnic disparities in prescribing long-acting atypical antipsychotics and an increased percentage of younger patients being treated with atypical depot antipsychotics over their oral equivalents.

## Introduction

Long-acting injectable antipsychotic medications (LAIs) have been proven to be both safe and effective for patients suffering from schizophrenia [1]. LAIs carry additional benefit in ensuring medication delivery among patients with surreptitious non-adherence. In patients with schizophrenia, non-adherence rates are estimated to be in the range of 40-50% [2]. Clinical Antipsychotic Trials of Intervention Effectiveness (CATIE) revealed a 74% rate of discontinuation of oral antipsychotics within six months of use, necessitating the role of depot antipsychotics [3]. LAIs also relieve the burden of taking daily medications, prevent complete loss of effectiveness for missed doses, and enable clinicians to track noncompliance [3-4].

Various reports have revealed racial disparities amongst those receiving LAIs [5-7]. For instance, individuals from ethnic minority groups were less likely than Caucasians to receive atypical LAIs for schizophrenia or other debilitating psychiatric illness [5]. A meta-analysis demonstrated that although the overall use of LAIs was comparable across ethnic groups, Latino and black patients were less likely than non-ethnic minorities to be treated with second-generation LAIs [7]. Eliminating any racial or ethnic disparities should remain a national goal for prescribing clinicians.

Many patients treated during their first episode of schizophrenia respond favorably to antipsychotics and can achieve appreciable levels of symptom remission within the first year [8]. The time period during which patients manifest psychotic symptoms prior to treatment, known as the duration of untreated psychosis (DUP), has been found to be associated with poor clinical outcomes [9]. Rapid implementation of LAIs has been shown to lessen the severity and duration of acute psychotic exacerbations [10]. Unfortunately, LAIs are generally underutilized, raising concern about whether target age demographics are being adequately addressed [11].

This study aims to compare risperidone injectable and paliperidone Palmitate (two atypical long-acting injectable antipsychotics), with risperidone oral to determine whether there lies a disparity with prescribing atypical LAIs within ethnic or age groups amongst a county inpatient population. Furthermore, since atypical LAIs have been shown to be efficacious for first-episode psychosis, we sought to examine whether the highest-risk age range (18 - 34 years old) was being targeted with these long-acting formulations.

## Materials and methods

The study sample consists of all patients aged 18-64 years receiving either risperidone injectable or paliperidone palmitate (r-LAIs), and risperidone oral across four years (2013 – 2017) within the Los Angeles County Department of Health Services (LAC DHS) electronic health record from a combined pool of 63,134 total inpatient visits. The LAC DHS is a regional, integrated system of providers, clinics, and hospitals that delivers quality, community-based care. The basic sampling unit is the physician-patient encounter or visit. This research used pre-existing, de-identified data and was deemed exempt research involving human subjects by the LAC DHS.

Data collected over a four-year period included patient ethnicity, age range, and use of r-LAIs and risperidone oral. Individuals were sub-classified into the following categories: age (18-34, 35-44, 45-54, and 55-64 years) and ethnicity (Asian, black, white, and other). This data was analyzed using chi-squared testing to compare ethnicity and age with the use of r- LAIs versus risperidone oral. Data were entered and analyzed using IBM SPSS Statistics v. 21.0 (Armonk, NY).

## Results

Among the 63,134 total inpatient visits, there were 3,011 patient visits that included the use of an atypical antipsychotic. Of these 3,011 visits, 452 (15.0%) were on r-LAIs. Of this group, 281 (62.2%) were male and 171 (37.8%) were female. Of the 3,011 visits, 2,559 (85.0%) were on risperidone oral. Of this group, 1,683 (65.8%) were male and 876 (34.2%) were female.

Table 1 summarizes the use of r-LAIs or risperidone oral amongst different ethnicities and ages. No statistically significant disparities were identified with the use of r-LAIs as compared to oral risperidone amongst ethnic groups (chi-square = 0.88, df = 3, p = 0.831), however, there was a statistically significant difference with the use of r-LAIs as compared to oral risperidone amongst age groups, favoring younger patients (chi-square = 13.46, df = 3, p < 0.004).

**Table 1:** Use of paliperidone palmitate or injectable risperidone versus oral risperidone amongst different ethnicities and ages

	Paliperidone palpitate or injectable risperdone (N = 452)		Oral risperdone (N = 2559)				
	Number of patients	% of patients	Number of patients	% of patients	chi-square	df	P
Ethnicity							
Asian	21	4.6	101	3.9	0.88	3	0.831
Black	115	25.4	649	25.4			
Other	182	40.3	1008	39.4			
White	134	30.0	801	31.3			
Age Range (years old)							
18-34	235	52.0	1099	42.9	13.46	3	0.004
35-44	69	15.3	501	19.6			
45-54	84	18.6	522	20.4			
55-64	64	14.2	437	17.1			

## Discussion

This study provides recent estimates of how r-LAIs were prescribed as compared to oral risperidone amongst different ethnicities in a large county inpatient system. Our findings are in disagreement with previous studies [5-7]. Reports have indicated that ethnic minorities are less likely to receive optimal access to atypical LAIs [7]. However, our data shows that there were no significant racial or ethnic differences in how these medications were prescribed. Each ethnicity was just as likely to receive r-LAIs as they were to receive oral risperidone. As racial disparity may affect the quality of care, subsequent studies should examine whether racial parity on receiving long-acting injectable antipsychotics improves the quality of care [12]. Particularly, our data indicate that both Asian Americans and African Americans are as likely to receive optimal care, as defined by likelihood, to receive depot antipsychotics in a large county inpatient system.

This study also provides recent estimations of r-LAI use as compared to oral risperidone amongst different age ranges. Historically, LAIs have been reserved for patients with the poorest compliance and for those in later stages of the disease [13]. However, our data report a higher percentage of individuals aged 18-34 years (in earlier stages of the disease) being treated with atypical LAIs over oral Risperidone. Interestingly, recent studies have indicated that LAIs can be effective even during first-episode psychosis [7-9]. For such patients, LAIs were associated with an improved quality of life and a clinically irrelevant incidence of extrapyramidal side effects [7]. The higher percentage of individuals on r-LAIs (52.0%) over oral risperidone (42.9%) within this age range (18-34 years of age) may illustrate an emerging trend to address early psychosis with newer, long-acting formulations.

This study has several limitations. First, there is a lack of data regarding other atypical LAIs or oral paliperidone, as these medications were not available on the formulary. Second, there is a lack of generalizability of these results towards other counties; this study only used de-identified data of one county. Third, the study did not account for other factors that contribute to racial disparities due to a lack of individual-level data. Fourth, there is a lack of data regarding psychiatric diagnoses as well as co-morbid substance use disorders in this study population [14-15]. Finally, although Los Angeles County has a large Hispanic population, the LAC DHS dataset does not have a separate racial category for the Latino population. This makes it impossible to calculate a reliable percentage of the Hispanic population for this particular study.

## Conclusions

Our data indicate a lack of ethnic disparities (particularly for African and Asian Americans) in prescribing atypical long-acting injectable antipsychotics and an increased percentage of younger patients being treated with atypical depot antipsychotics over their oral equivalents. As LAIs are both safe and effective for patients with schizophrenia, the results of this study indicate that quality of care can be achieved in a community-based psychiatric inpatient population. Additional methodologically rigorous studies using standardized outcome measures are needed to examine the relationship between the utilization of LAIs and the quality of care.
